# A Fault Tolerant Surveillance System for Fire Detection and Prevention Using LoRaWAN in Smart Buildings

**DOI:** 10.3390/s22218411

**Published:** 2022-11-01

**Authors:** Abdullah Safi, Zulfiqar Ahmad, Ali Imran Jehangiri, Rohaya Latip, Sardar Khaliq uz Zaman, Muhammad Amir Khan, Rania M. Ghoniem

**Affiliations:** 1 Department of Computer Science and Information Technology, Hazara University Mansehra, Mansehra 21120, Pakistan; 2 Department of Communication Technology and Network, Universiti Putra Malaysia (UPM), Serdang 43400, Malaysia; 3 Department of Computer Science, COMSATS University Islamabad, Abbottabad Campus, Abbottabad 22060, Pakistan; 4 Department of Information Technology, College of Computer and Information Sciences, Princess Nourah bint Abdulrahman University, P.O. Box 84428, Riyadh 11671, Saudi Arabia

**Keywords:** Internet of Things, LPWAN, LoRaWAN, fire detection, response time

## Abstract

In recent years, fire detection technologies have helped safeguard lives and property from hazards. Early fire warning methods, such as smoke or gas sensors, are ineffectual. Many fires have caused deaths and property damage. IoT is a fast-growing technology. It contains equipment, buildings, electrical systems, vehicles, and everyday things with computing and sensing capabilities. These objects can be managed and monitored remotely as they are connected to the Internet. In the Internet of Things concept, low-power devices like sensors and controllers are linked together using the concept of Low Power Wide Area Network (LPWAN). Long Range Wide Area Network (LoRaWAN) is an LPWAN product used on the Internet of Things (IoT). It is well suited for networks of things connected to the Internet, where terminals send a minute amount of sensor data over large distances, providing the end terminals with battery lifetimes of years. In this article, we design and implement a LoRaWAN-based system for smart building fire detection and prevention, not reliant upon Wireless Fidelity (Wi-Fi) connection. A LoRa node with a combination of sensors can detect smoke, gas, Liquefied Petroleum Gas (LPG), propane, methane, hydrogen, alcohol, temperature, and humidity. We developed the system in a real-world environment utilizing Wi-Fi Lora 32 boards. The performance is evaluated considering the response time and overall network delay. The tests are carried out in different lengths (0–600 m) and heights above the ground (0–2 m) in an open environment and indoor (1st Floor–3rd floor) environment. We observed that the proposed system outperformed in sensing and data transfer from sensing nodes to the controller boards.

## 1. Introduction

Over the past few years, IoT has become an important and quickly growing technology. It is a communications infrastructure composed of equipment, buildings, electrical systems, modern vehicles, and everyday things [1]. These devices link to the Internet to gather and distribute data consistently, enabling remote access and management. The “IoT” was introduced in 1998 by Kevin Ashton [2] and has since been defined as the way smart things work together and talk to each other [3]. The IoT-enabled smart devices are utilizing emerging applications and services to benefit the community in education, infotainment, structural and traffic surveillance, and the healthcare sector. These devices include but are not limited to sensors, actuators, Radio Frequency Identification (RFID) tags, smartphones, smartwatches, and Body Area Networks (BAN) devices. Moreover, those devices can sense, collect, process and transfer the data in the IoT paradigm, meeting the basic needs of end users [4].

In studies [5,6], IoT devices connected to the Internet are projected to grow by over one trillion by 2025, endeavoring Smart Cities, smart grids, smart homes, e-healthcare, e-banking, and logistics [7]. Hence, it becomes more challenging to maintain the optimal standards of security, safety, reliability, latency, user mobility, throughput utilization, and fault tolerance for the effective usage of IoT-empowered devices and applications. Regarding safety, fire suppression is vital in protecting human lives, structures, and IT assets. Much attention has been paid to Fire Detection Systems (FDS), which have reduced the risk of fire to people and property. False alarms, however, are quite losable if the occurrence takes place in a commercial facility.

Furthermore, false fire alarms can be a hassle for the fire department, resulting in resource shortages and unneeded disruption that causes panic. Additionally, traditional fire suppression systems lack the IoT’s intelligence and are hardcoded [8]. Communication technologies support IoT networks for short-range sensing applications, including RFID, Bluetooth, Wireless Local Area Network (WLAN), Near Field Communication (NFC), Low-Rate Wireless Personal Area Networks (LR-WPANs), and IPv6 over Low-power Wireless Personal Area Networks (6LowPANs). Here, Bluetooth and WLAN are suited for short-range and high-speed data transfers. LR-WPANs and 6LowPAN target the application areas requiring low data rates and short-range.

Low Power Wide Area Networks (LPWAN) are an emerging technology offering low-speed and long-range data transfers with minimal battery consumption compared to the earlier typical technologies [9]. LPWAN is a low-power, long-distance communication method used in Machine to Machine/Internet of Things (M2M/IoT) networks. Large-scale sensor-based industrial deployments are also influencing trends. To support such sensor installations in various situations, additional communication protocols which can coexist with current cellular communication systems, such as 3G, 4G, and 5G, and local standards, such as Bluetooth, and 802.11, are required. Popular communication technologies, such as Narrowband IoT (NB-IoT), SigFox, and Long Range Wide Area Networks (LoRaWAN), are utilized to lower the sensors’ power consumption while supporting long-range transmissions up to hundreds of meters or even a few kilometres [10,11]. One of the LPWAN technologies now popular in the IoT sector is LoRaWAN. Supporting devices with years of battery life becomes flexible for IoT networks in which end nodes transmit fewer sensing data over long distances and in dynamic environmental constraints. The LoRa networks can control many nodes in many locations with just one receiver, compared to WLAN-based systems that require several access points to expand the coverage area. Combining LoRa with Wi-Fi helps keep the price of establishing an Internet of Things system down [12,13]. As IoT-based architectures become more prevalent, there is a growing need for LPWAN, which can provide monitoring solutions in cases like fire risk assessments requiring coverage of a vast region [7].

The following are the highlights of the contributions made in this work.

- We propose a fault-tolerant surveillance system for fire detection and prevention in smart buildings exploiting LoRaWAN to reduce latency and enhance reliability;
- Using Wi-Fi LORA 32 sensors and board, we designed and developed a system that can detect smoke, natural gas, LPG, propane, methane, hydrogen, alcohol, temperature, and humidity to prevent fires caused by them;
- We performed extensive experiments over a distance up to 600 m long and up to 2 m high from the ground. The proposed system is tested over 50 packets of varying (16–64) bytes payloads sent/received;
- The performance evaluation is based on the Received Signal Strength Indicator (RSSI), network delay, and response time parameters;
- We demonstrated that LoRaWAN technology is ideal for deploying fire detection and prevention systems in large smart structures and successfully transmitting detected data.

The remainder of the paper is laid out as follows. The recent related works are covered in Section 2. The background of LPWAN and LoRa is explained in Section 3. In Section 4, the problem statement is formulated. Section 5 endeavours the research design, methodology, and real-time scenarios relevant to the experiments. The performance evaluation and results are discussed in Section 6. Finally, Section 7 concludes the paper with potential future research directions.

## 2. Related Work

Different features of LoRaWAN have been the subject of attention in various studies to develop fire detection and prevention surveillance systems. Some studies present experiments comprising fewer nodes with shorter ranges. Some works have proposed the architectures/algorithms and assessed their investigations in simulators. We discuss these studies in further detail as given.

According to Vega-Rodriguez, R. et al. in [7], forest fires are among the Mediterranean region’s most pressing environmental problems. There is a growing need for innovative solutions that leverage low-power, long-range networks and the IoT to control and monitor fires better. The research employed a cheap Long Range (LoRa) network to analyze fire risks and spot forest fires. The system includes a LoRa node and several sensors for measuring carbon dioxide levels, relative humidity, temperature, and wind speed. According to the 30-30-30 rule, the evaluation process must be completed in 30 min. Visitors to the website may see the nodes’ data collected in real-time. We conducted field tests to see how far this system might travel and found that it could cover up to 600 m.

The authors of [14] took home and workplace safety into account. Using WSN, they created a unique model. They included smoke and fire sensors and temperature and humidity sensors when developing the model. They presented earlier studies that show Wireless Sensor Networks (WSNs) can recognize fire alarms. A wireless sensor network with three sensors is to be established. To learn more about one’s home, an application was created. The Android Studio-programmed Arduino implant collects information from sensors, including gas, temperature, flame, and humidity. Let’s say it checks the gathered data and finds that the control levels are too high. When that happens, you may engage with the wireless network. Preventative fire monitoring is made possible by sending a notification alert message to users’ mobile devices. Without an internet connection, the system is inoperable.

To offer fast fire detection and alarm and status monitoring of fire-extinguishing abilities while spending the least electrical power, Wen-hui Dong created a specialized wireless communication protocol for fire detection and alarm and a comprehensive set of wireless automated fire alarm systems. There was no guidance on how to identify fires from the system. There is currently no false alarm detection built into any authentication methods [15].

Most applications of wireless communication technology may be found in the realms of building automation and centralized control. In [16], the authors proposed software enabled with a wireless sensor fire detection system. The system monitors the fire alarm from a large distance to assist the evacuation process; however, the control centre cannot respond quickly, affecting the response time. The reason for this is that it lacks the connectivity of the main server with all sensors. To prevent fires, the authors of [17] developed a wireless IoT-based fire alarm system that uses an ad hoc network with many nodes placed strategically around the house. Each of these nodes is equipped with a microcontroller from the company ESP8266 called a Node MicroController Unit (nodeMCU), coupled with sensors that detect smoke, humidity, temperature, flame, Carbon Monoxide (CO), and methane. Each node creates separate wireless networks. The central node communicates with the other nodes through a Raspberry Pi microcontroller and a 4G module. When a fire is detected, the node sends a signal to a centralized node, telephones the user, notifies the fire department, and triggers the local alarm system via SMS messaging. If you cannot go online or if your connection is poor, this strategy will not work as well for you. When a fire breaks out, sensors and the SigFox LPWAN protocol are deployed for accurate temperature and gas monitoring.

Ahmad Alkhatib [18] described a WSN system for forest fire detection utilizing a sub-network coverage method. The deployed network consisted of wasp motes with Zigbee transmitters. The proposed system divided the network into three subnetworks increasing the network’s lifetime and scalability by 2.7% and energy efficiency by 63% compared to the traditional fire detection systems. In [19], the authors examined the LoRaWAN’s effectiveness in an industrial setting. They considered the indoor environment of the flower industry as a use case. Using only one gateway and twenty-one end nodes, an area of 180 × 190 m, or around 34,000 square meters, was measured. The authors used a simulation developed in the Python programming language to examine LoRaWAN’s scalability across a wide industrial region. The study found that a single gateway could sustain up to 6000 end nodes if 75% of the nodes sent packets once each hour and the other 15% did so once every five minutes.

Sendra et al. [20] developed a technique to assess potential fire hazards using the LoRaWAN technology, although they did not describe the end node distribution over the coverage terrain. A LoRa node was fitted with multiple sensors to measure the desired system’s humidity, temperature, CO_2_, and wind speed. To determine the coverage area, the work began by computing the RSSI and Signal-to-Noise Ratio (SNR) for many locations in the investigated area in Motril, Spain. After that, the ability of a single node to identify several characteristics was evaluated for 20 h, with measurements were taken every 28 min. The results indicate that the proposed method can span a 4-km radius with a single gateway. Throughput, latency, power consumption, and packet loss ratio were also analyzed to determine the efficacy of LoRaWAN networks co-located with 5G networks [21]. The experimental setup includes two Raspberry Pi 2 nodes, a gateway server, and a client device. These components were all placed at various locations within the same building, ranging from 50 cm to 60 m.

LoRaWAN’s low power consumption makes it a prominent technology. It is also meant to improve capacity and coverage while reducing costs. Using a realistic network model, researchers in [9] investigated the viability and scalability of LoRaWAN in the Mina region, a valley situated east of Makkah City. The place is known as the world’s largest tent city. It can house up to 3 million pilgrims annually and contains more than 100,000 tents. The OMNeT++ simulator and Flora model were used to run extensive simulations to examine the delivery ratio, collision, and SF dispersion for the simulated scenario with up to 10,000 end devices. The simulations demonstrated the constancy and dependability of LoRaWAN’s high success rate. Table 1 presents a comparison of some significant existing works.

## 3. Background of LPWAN and LoRa

“LPWAN” refers to technologies that link low-power devices with sensors and controllers in IoT applications. IoT communications require great range, long battery life, and affordable endpoints. [22,23]. These factors establish its two important properties: low power budget and large transmission range. It also imparts low-cost, and low-data-rate IoT needs. It allows rural communication up to 40 km and urban communication from 1 to 5 km [14]. In this context, “low-power wide-area network” (LPWAN) refers to technologies that use either licenced or unlicensed spectra and might be either private, proprietary, or open standard. Engineers and scientists utilize this to implement WSNs for Internet of Things applications depending on price, range, and power consumption [9]. Short-range, high-bandwidth networks such as Bluetooth and Wi-Fi are combined with cellular networks in LPWAN. LoRa is free software that operates in ISM sub-GHz bands. Chirp Spread Spectrum (CSS) modulation allows for reliable decoding of LoRa transmissions even when the signal strength is below the noise threshold. LoRa is less complicated to implement than SigFox and NB-IoT since it uses unlicensed airwaves, relies on open-source infrastructures, and requires less money to get up and running [24,25].

LoRa technology has allowed Internet of Things (IoT) applications to progress even further. In contrast to Wi-Fi-based systems, which need many access points to increase coverage area, the LoRa network can handle numerous nodes around the area with just one receiver. By merging LoRa with Wi-Fi technology, the cost of setting up an IoT system decreases. The authors of [12,26] examined the real-world implementation of an IoT system using LoRa and Wi-Fi technology. The Chirp Spread Spectrum (CSS) modulation scheme in LoRa protects a signal from channel noise and security [12,26].

## 4. Problem Statement

The current monitoring systems seek to identify a fire in indoor and outdoor settings to lessen risk in the impacted areas by sending out prompt and trustworthy alerts [27,28]. However, these solutions largely depend on WLANs to cover larger distances. The WLANs have built-in limitations such as range, stability, collisions, power consumption and response time, degrading the fault tolerance capability in critical safety systems like fire detection and prevention. We propose a novel fire detection and prevention system with intelligent surveillance and fault tolerance capability to overcome the limitations.

## 5. Research Design and Methods

**Figure 1:**
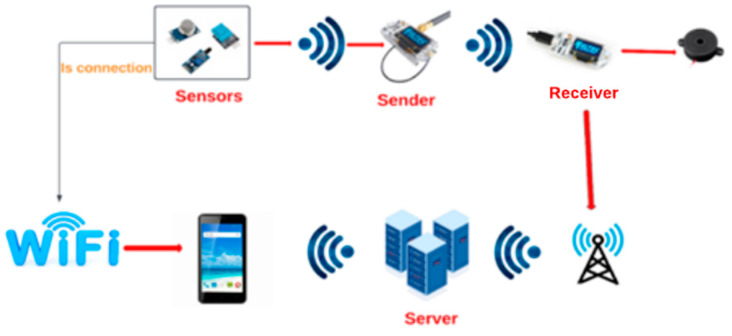
Proposed F2DPS System.

This study introduces a robust and reliable fire detection and prevention surveillance system for intelligent structures. It is a Fault-tolerant Fire Detection and Prevention Surveillance (F2DPS) system. We employed both Wi-Fi and LoRaWAN as wireless communication technologies, with Wi-Fi serving as the primary means of data delivery in areas where it is available and LoRaWAN filling in the gaps elsewhere. The electricity may be shut off in the event of a fire, but the LoRaWAN network would continue to function. LoRaWAN, which consists of Wi-Fi, LoRa, and (Bluetooth Low Energy) BLE, is utilized by the Heltec Wi-Fi LoRa 32 V2 (HELTEC AUTOMATION, Chengdu, China) development platform. One Heltec Wi-Fi LoRa 32 V2 device was utilized for sending information, and another for receiving it. When a signal is picked up by the LoRaWAN sensor and sent to the LoRaWAN sender board, the data is sent to the receiver. We can get a sense of how a smart building’s fire detection and prevention monitoring system is set up from Figure 1.

A campus that uses smart technology will have a fire detection and prevention system to handle false alarms. Wi-Fi and LoRaWAN were employed as the transfer mechanisms for the detected data. Wi-Fi will be utilized for communication at first, and if that fails to work, LoRaWAN will be used instead. The grove-gas sensor (MQ2) and infrared radiation (IR) flame sensor can detect air fires using methane, hydrogen, smoke, propane, alcohol, and carbon monoxide.

Several existing systems are aimed at detecting fire in outdoor and indoor places to reduce the risk in the affected areas by sending out timely and reliable alarm messages. However, the existing solutions rely on a single communication technology, such as the Wi-Fi network. There are various places in a Wi-Fi network where communication occurs at bottleneck nodes, and there is no fault tolerance. The failure of bottleneck nodes makes the whole system ineffective. Therefore, the proposed research work is important in addressing the above issues.

### 5.1. Sensing Data Acquisition and Sensor Selection

**Figure 2:**
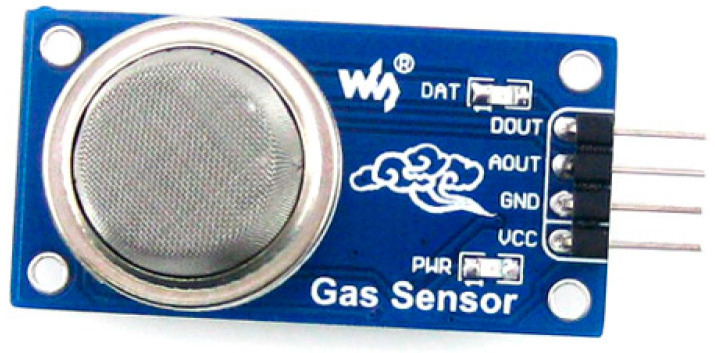
MQ2 Gas Sensor.

Since the Grove-Gas Sensor (MQ2) module is great for spotting gas leaks; we used that along with an infrared flame sensor to collect data (home and industry). It can detect hydrogen, liquefied petroleum gas, carbon monoxide, alcohol, smoke, and propane [24]. Measurements may be taken with it because of its high sensitivity and fast response time. Utilizing the Infrared (IR) flame sensor, we can locate a fire or any infrared source. It is possible to pick up the light of a flame or other source with a wavelength between 760 and 1100 nm, which could help put out fires or be used in a heat detector, as shown in Figure 2.

### 5.2. Wi-Fi Lora 32 Board

**Figure 3:**
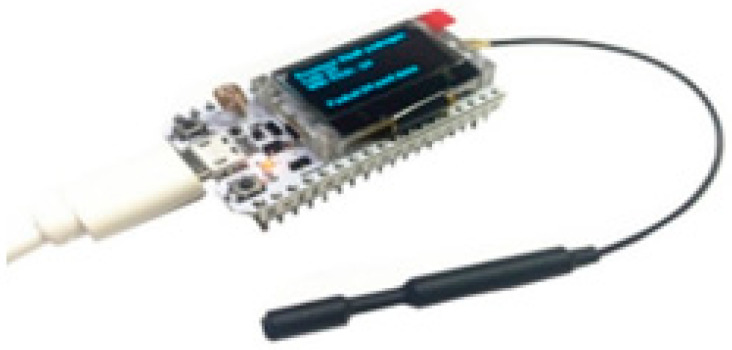
Heltec Wi-Fi LoRa 32 V2.

HeltecWi-Fi-LoRa 32: The Wi-Fi LoRa-32 is an IoT-focused device developed by the Heltec group Heltec Automation (HELTEC AUTOMATION, Chengdu, China). The development board’s functionality is built on the ESP32 microcontroller, while an integrated SX1278 chip handles LoRa connectivity, as shown in Figure 3. These two gadgets can communicate using the SPI interface [29]. Although the Heltec device is designed to be a sensor node, it may also be configured as a LoRa-WAN gateway that connects to the server via the TCP/IP protocol. This restricts its capabilities and makes it impossible to transmit data to sensor nodes on the downlink.

### 5.3. Dataflow Model for Data Reading and Transmission

**Figure 4:**
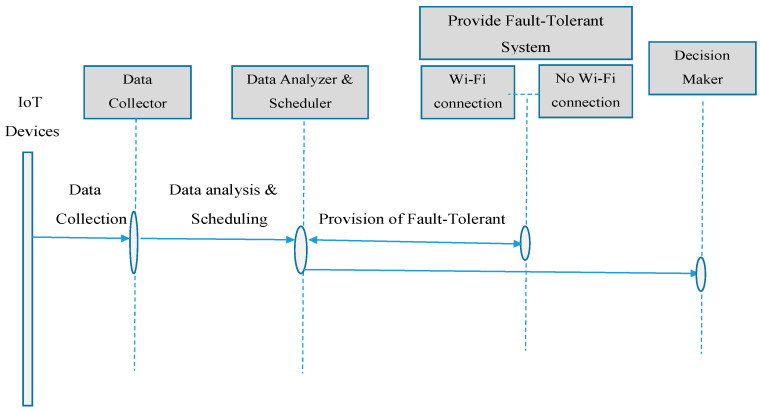
Dataflow model.

Two Heltec Wi-Fi LoRa 32 V2 boards are used in the project, one as a transmitter attached to an MQ2 Gas sensor and the other as a receiver attached to a Light Emitting Diode (LED). The transmitter LoRa board will transmit a LoRa packet with a string-based message whenever gas or smoke is detected. The signal transmitted to an LED is decided using this string comparison when the receiver receives the packet; if it is processed, it is compared to a specified text. In Figure 4, the dataflow model is displayed. Collected sensor data through IoT devices/nodes, which is heterogeneous and is executed/evaluated on cloud resources.

As a result, there is a risk of an internet connection failure in smart buildings. The authors do not consider all these deficiencies in the existing solutions, which rely on a single communication technology such as the Wi-Fi network. There are various places in a Wi-Fi network where communication occurs at bottleneck nodes, and there is no fault tolerance. The failure of bottleneck nodes makes the whole system ineffective. There is no appropriate data management and fault-tolerant mechanism for fire detection tasks and IoT devices. If Wi-Fi fails or some nodes cannot send data timely and efficiently, the response time and latency will be maximized. Therefore, the F2DPS scheme will be an efficient solution for fault-tolerant aware data management and reduce response time and latency for fire detection IoT in smart buildings.

### 5.4. Real-Time Testing

In this phase, we assessed our work in a practical setting. The proposed research project is evaluated using two performance evaluation criteria: (a) Response Time and (b) Network Delay.

#### 5.4.1. Response Time

This is the time it takes for data to move between two network nodes. All sorts of delays, including processing and network delays, are added to determine the response time. Milliseconds (ms) are used to quantify response time [30]. The Equation (1) is used to compute the network delay:

(1) $$RES_{T}=P_{D}+N_{D}$$

where the *RES_T_* expresses the response time, *P_D_* denotes the processing delay, and *N_D_* denotes the network delay.

#### 5.4.2. Network Delay

The network delay is calculated by combining all types of delays, including transmitting and receiving delays. Milliseconds measure the network delay in ms [30]. The Equation (2) is used to compute the network delay:

(2) $$N_{D}=REC_{T}-S_{T}$$

where the *N_D_* expressed the network delay, *REC_T_* denotes the receiving time, and sending time is denoted by *S_T_*.

## 6. Performance Evaluation

We will briefly outline the method and system components connected to results, then discuss the consequences of employing the system structure in a real-world setting. This test was conducted to determine the maximum range, network latency, and reaction time over which a LoRa device can receive LoRa packets and compare the results to competing technologies like Wi-Fi. This experiment compares LoRaWAN’s performance in an open environment with a closed one.

### 6.1. Data Gathering

We were able to transmit MQ2 sensor data to the receiver and gather data on two measurement parameters: response time and network latency, using the Wi-Fi Lora 32 Development Board. We also discussed the measurements and location where the 433E6 Hz frequency data was gathered in this experiment. Data collection took place at numerous campus locations to represent various situations, including an open space unencumbered by structures or other barriers. The confined area inside a building is more impacted by other blocks and barriers distorting the wireless propagation. For indoor modelling, we chose a square-shaped building of three floors with a wall thickness of six inches, a height of ten feet, and several rooms on each Floor. Experiments were performed on each Floor with a variation in payload size.

### 6.2. Data Analytics and Measurement Circumstances

The communication range, network delay, and reaction time that the Lora device can receive LoRa packets are all described in this section. The measurements are conducted in two distinct environments: an open space and a closed location.

#### 6.2.1. Response Time Measurements in Open Area

**Figure 5:**
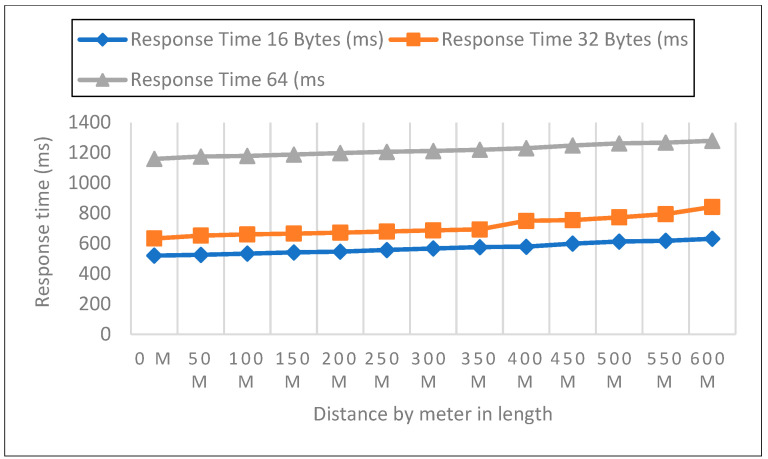
0-m height and 16, 32, 64 bytes payload size.

**Figure 6:**
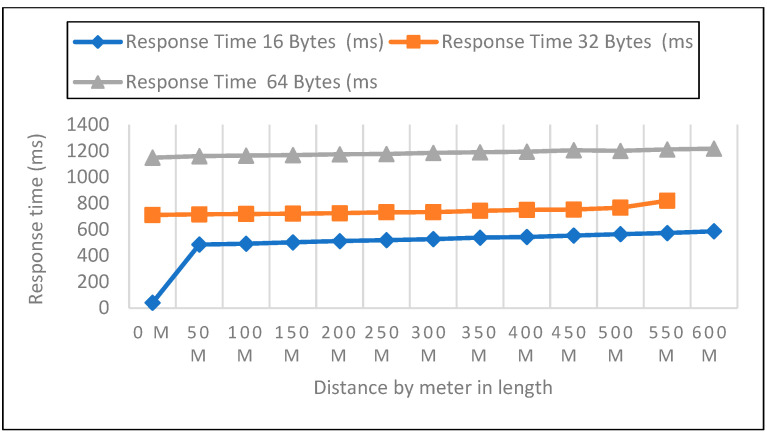
A height of 1-m and 16, 32, 64 bytes payload size.

**Figure 7:**
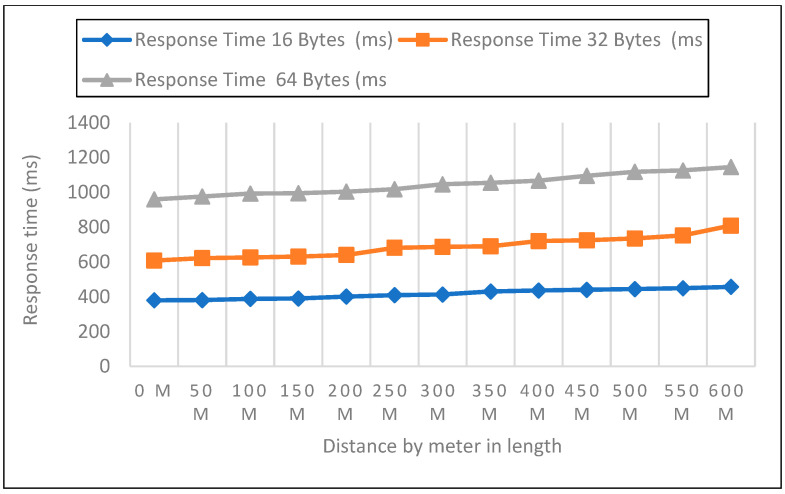
A height of 2-m and 16, 32, 64 bytes payload size.

Measurements were collected in an open setting inside the campus. The sender node is located one meter above the ground and two meters above the ground. The distance in this experiment varied from 0 to 600 m. 50 data packets with varied payloads of 16, 32, and 64 bytes were then sent when each node was configured for LoRa, as shown in Figure 5, Figure 6 and Figure 7. In each experiment, 50 packets were sent at a distance increase of 50 m; the packet received, RSSI, network delay, and response time were recorded. Additionally, we compared the three payload sizes with each meter’s height.

- Response Time Analysis

Board for LoRa Development Sent packet from the sensor to the development board of the receiver calculated by Equation (3) and represented in Table 2.

(3) $$Response_{Time}=P_{D}+N_{D}$$

B. Network Delay Analysis

**Figure 8:**
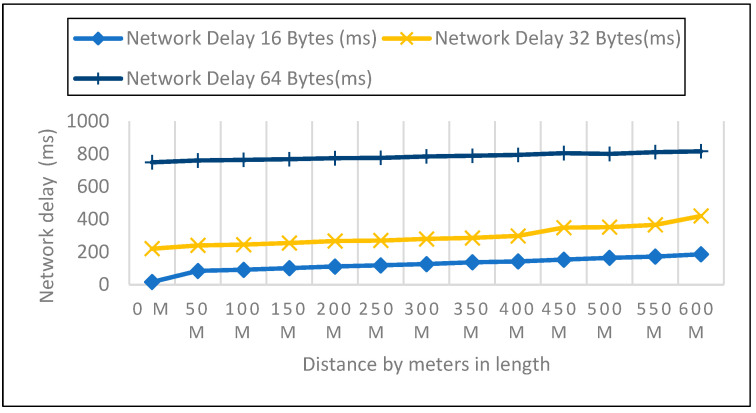
A height of 0-m and (16, 32, 64) bytes Payload Size.

**Figure 9:**
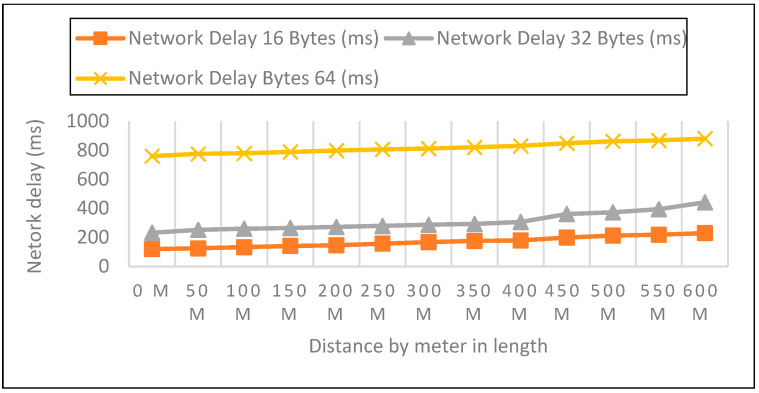
1-m height and (16, 32, 64) bytes Payload Size.

**Figure 10:**
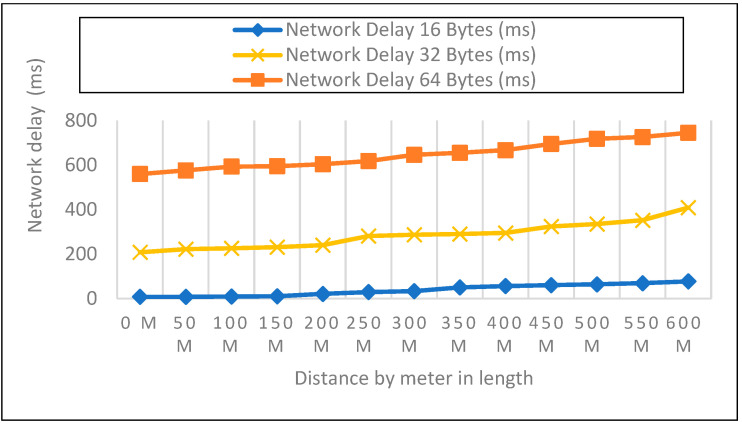
2-m height and 16, 32, 64 bytes Payload Size.

We looked at the performance of LoRaWAN in an open environment so that we could determine the response time over which a LoRa device could receive LoRa packets. The sender node can be found on the ground, at an elevation of 0 m, 1 m, and 2 m above the ground, as shown in Figure 8, Figure 9 and Figure 10. During this particular experiment, the distance varied anywhere from 0 to 600 m. After the LoRa configuration had been completed for each node, 50 data packets with variable payloads of 16, 32, and 64 bytes were transmitted. Table 3 represents the overall network delay in different scenarios.

We evaluated the performance of LoRaWAN in an open environment so that we could determine the amount of network latency that a LoRa device is able to withstand. In a field setting, the experiment covered a distance range of zero to six hundred meters. After configuring each node for LoRa, 50 data packets with variable payloads of 16, 32, and 64 bytes were transmitted. The bytes in each payload increased by an increment of 2. We delivered 50 packets in each experiment with a 50-m elevation increase and recorded the network delay. Additionally, we contrasted the three payload sizes with each meter’s height.

#### 6.2.2. Response Time Measurements in Closed Area

**Figure 11:**
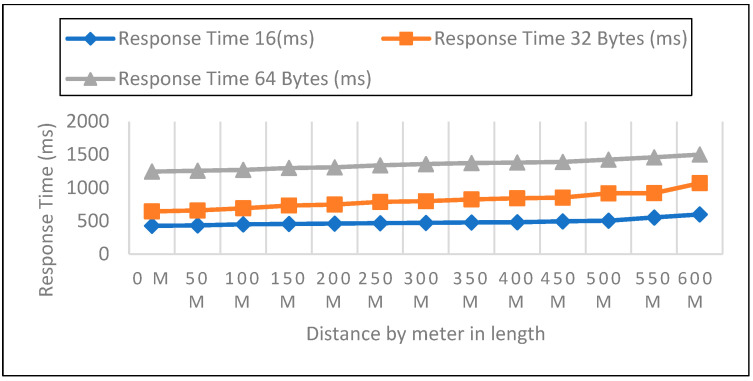
First Floor with 16, 32, 64 bytes Payload Size.

**Figure 12:**
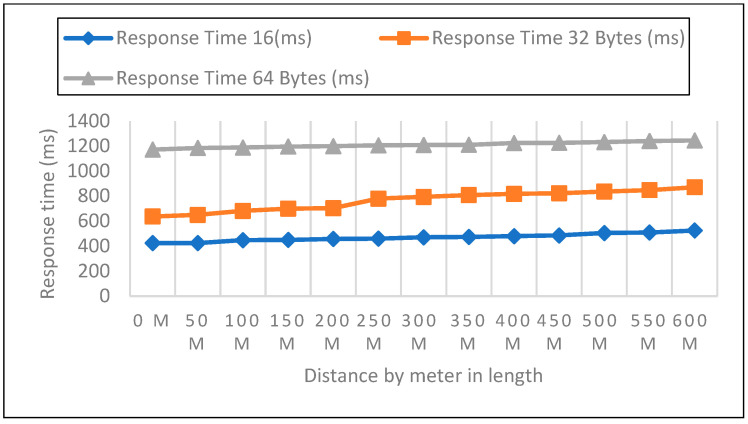
Second Floor with 16, 32, 64 bytes Payload Size.

**Figure 13:**
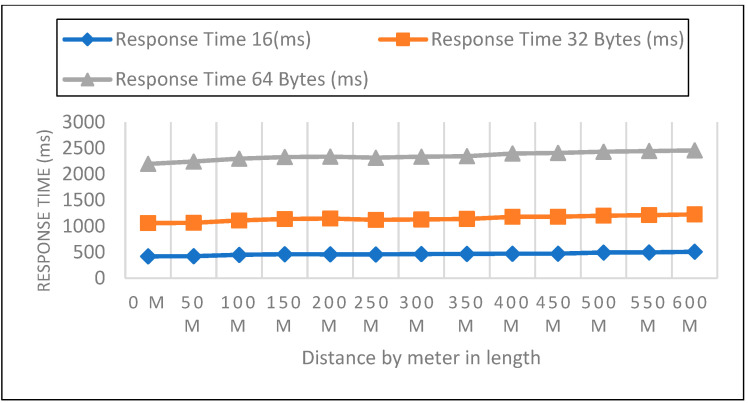
Third Floor with 16, 32, 64 bytes Payload Size.

Measurements were carried out in a safe area inside the institution. The sender node was located on the first, second, and third floors at three different heights. The distance in this experiment varied from 0 to 600 m. Then, 50 data packets with varied payloads of 16, 32, and 64 bytes were transmitted when each node was set up for LoRa. In each experiment, 50 packets were sent at a distance increase of 50 m; the packet received, RSSI, network delay, and response time were then recorded, as shown in Figure 11, Figure 12 and Figure 13. In a closed environment, we measured response times on the first, second, and third floors using various payload sizes. Table 4 demonstrates the calculated response time with different payloads.

To assess how quickly a LoRa device can receive LoRa packets, we examined the performance of LoRaWAN in a sealed environment. Three heights are represented by the sender node: one, two, and three floors above sea level.

#### 6.2.3. Network Delay Analysis for Closed Area

**Figure 14:**
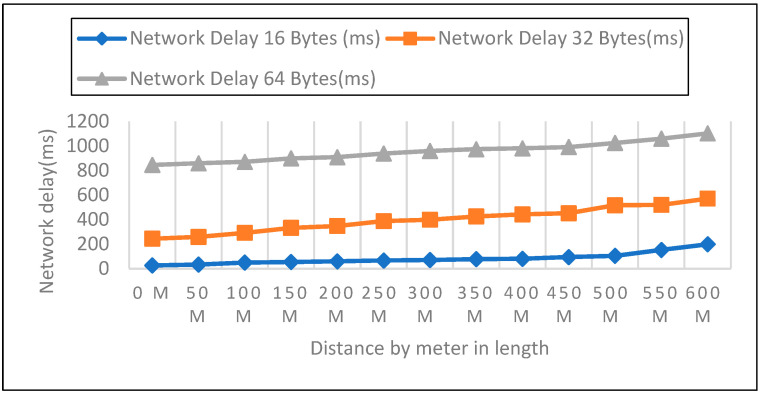
Network Delay Compression Analysis: First Floor with 16, 32, 64 Payload Size.

**Figure 15:**
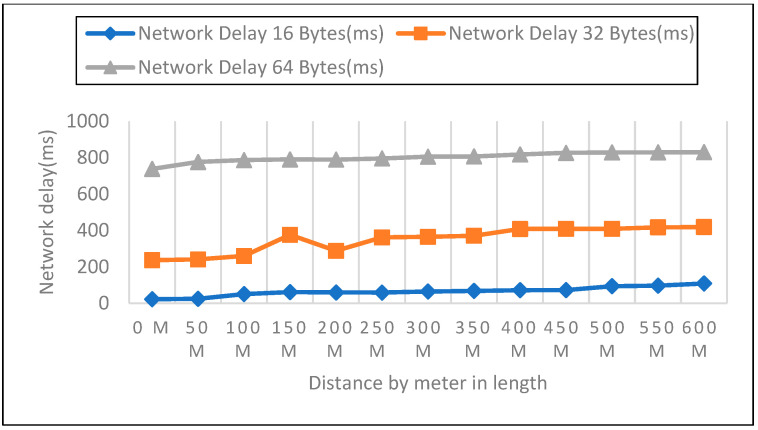
Second Floor with 16, 32, 64 Payload Size.

**Figure 16:**
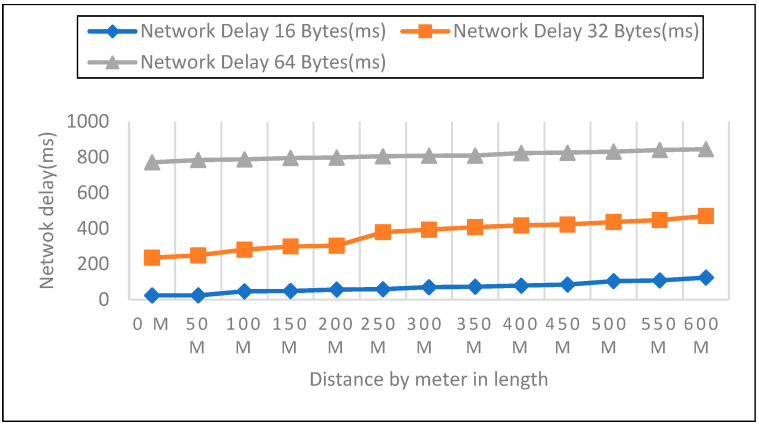
Third Floor with 16, 32, 64 Payload Size.

We measured network latency in the enclosed space on the first, second, and third floors using three different payload sizes, as shown in Figure 14, Figure 15 and Figure 16. Comparison of the other network performance parameters with respect to payload and height is displayed in Table 5.

As part of the experiments, the performance of LoRaWAN in a closed environment was analyzed so that the reaction time over which a LoRa device may receive LoRa packets could be determined. The sender node can be found on the bottom level, as well as on the first, second, and third stories above the ground level. In the course of this trial, the distance ranged anywhere from zero to six hundred meters. After configuring each node for LoRa, 50 data packets with variable payloads of 16, 32, and 64 bytes were transmitted. The bytes in each payload increment by 2. We sent 50 packets for each trial with a 50-m elevation increase and timed the responses. In addition to this, we compared the height of each meter with the three different payload sizes.

The analysis in performance of the proposed research work has been adopted from the benchmark proposed in the study [7,9,20]. The proposed research work has been tested in a real-world environment. We validated the work by evaluating the two parameters of response time and network delay. We used the Wi-Fi Lora 32 board for data transmission with 16-, 32-, and 64-bit payload sizes and the results were compared with different heights. We also performed experiments in open environments and in environments with obstacles such as buildings and trees.

False fire alarms can also cause problems for the fire department by using up resources unnecessarily and causing unnecessary disturbances that spread fear. We relate the false detection to the quality of data transmission and RSSI value. As the RSSI value approaches zero, the quality of data transmission is better, and in terms of RSSI value, the LoRaWAN technology is an excellent candidate for installing fire detection and prevention systems in smart buildings.

## 7. Conclusions and Recommendation

This study recommends designing a robust fire detection and prevention surveillance system for use in smart buildings, which would significantly reduce response times and latency. With the help of the Wi-Fi LORA 32 development board, we could integrate LoRaWAN technology. We devised the network and put the sensors in place to identify various combustible substances. The development board module provided the functionality of the transceiver. The transmitter board initiates communication with the Wi-Fi Lora 32 board receiver as soon as it determines that fire symptoms have been detected. In addition, a single receiver can receive signals sent out by several different transmitters due to this effort. Multiple experiments were carried out on sensors set up at various distances and heights from the ground’s surface. We sent out fifty packets and measured their reception, the RSSI, the network delay, and the response time. The testing results determined that the LoRaWAN technology is an excellent candidate for installing fire detection and prevention systems in smart buildings.

During the experimental analysis, it was found that when there was very cold weather or on a rainy day, the RSSI value was closer to zero. This indicates that the data transmission quality was better during the cold weather as compared to normal or hot weather. As a result, it can also be related to low data transmission quality with extremely hot temperatures, or a decision of fire detection can be made with very low data transmission quality based on extremely hot temperatures.

As a potential next step in the study, we plan to investigate how the LoRaWAN technology affects the energy it uses and its level of safety. Additionally, examining the performance of LoRaWAN in topological topologies with self-organizability and configurability, such as mesh networks, is an attractive field of investigation that can detect fires and decrease losses more effectively.

## Figures and Tables

**Table 1:** Comparison of the significant literature.

Authors and Year	Environment	Technology	Contributions	Limitations
Roberto Vega-Rodríguez et al. [7], 2019	Low-Cost LoRa-Based Forest Fire Detection Network	LoRa	The system is composed of a LoRa node and a set of sensors.	Less Receiver sensitivity indoors and in smart buildings.
M. Al Mojamed [9], 2022	Smart mina: LoRaWAN technology for smart fire detection	LoRa	Simulated a Flora model in OMNET++. Considered the packet delivery ratio and collisions.	Not a real-world fault-tolerant framework.
K. c et al. [10], 2017	Design and implementation of the mobile Fire Alarm System (FAS)	Wi-Fi	The Android Studio-coded Arduino implantation.	Lack of an internet connection renders the machine inoperable.
W. H. Dong et al. [15], 2016	Design of Wireless Automatic FAS	Wi-Fi	To achieve rapid energy-efficient fire detection, alarm, and governmental oversight of fire-fighting infrastructure.	The system did not identify flames. No authentication system detects false alerts.
Y. Liu and M et al. [12], 2016	Fire Monitoring System-Based ZigBee-Wi-Fi Networks	ZigBee-Wi-Fi Networks	Monitoring the fire alarm remotely, aiding in evacuation.	Model cannot connect to all fire-affected devices, and the monitor is not connected to a central server.
F. A. Saputra et al. [17], 2017	Early FDS for Home	Raspberry Pi	Typical monitoring of fire alarms.	Involves higher power consumption.
Ahmad Alkhatib et al. [18], 2016	Forest fire detection WSN system	Zigbee	Proposed a scalable system with three sub-networks.	Power hungry and no-fault tolerance.
Sendra et al. [20], 2020	fire risk assessment system based on LoRaWAN technology	LoRaWAN	The LoRa node has humidity, temperature, CO2, and wind speed sensors.	End node distribution and coverage are not defined.

**Table 2:** Response Time at varying sensor heights from the ground (0–2 m).

Size	16 Bytes						32 Bytes						64 Bytes					
Height	0 M		1 M		2 M		0 M		1 M		2 M		0 M		1 M		2 M	
Distance	R-T	RSSI	R-T	RSSI	R-T	RSSI	R-T	RSSI	R-T	RSSI	R-T	RSSI	R-T	RSSI	R-T	RSSI	R-T	RSSI
0	520	−22	40	−41.3	380	−48.16	633	−27.42	620	−24.88	608	−42	1160	−35.18	1149	−56.6	960	−55
50	525	−80.98	484	−99.06	381	−85.86	652	−92.82	640	−78.82	622	−84	1175	−99.8	1160	−76.86	976	−77.1
100	533	−92.3	491	−108	388	−86.58	660	−99.24	645	−89.82	626	−96.48	1179	−104.54	1164	−91	993	−86.72
150	541	−91.6	501	−109.8	390	−89.8	665	−94.84	655	−86.32	631	−92.8	1188	−92.48	1168	−93.52	995	−90.66
200	546	−107	511	−110.42	401	−103	672	−99.98	720	−92.52	640	−92.42	1198	−102	1174	−86.44	1004	−94.96
250	557	−103	518	−112.84	409	−104.54	679	−111.62	675	−97.5	681	−106.66	1206	−106.34	1176	−104.32	1018	−105.74
300	567	−109	526	−117.28	413	−113.38	687	−111.64	680	−100.66	687	−111.18	1212	−121.92	1185	−107.9	1046	−106.54
350	576	−115	537	−124.26	430	−113.96	693	−114	686	−108.38	690	−115.88	1220	−120.2	1189	−115.72	1055	−114.72
400	579	−118	542	−127	436	−117.48	706	−116.6	698	−106.28	695	−112.88	1230	−127.42	1194	−118.14	1067	−121.16
450	599	−116	553	−124.1	440	−118.2	730	−115.66	749	−111.86	724	−117.72	1248	−131	1205	−118.8	1095	−111.42
500	613	−123	564	−128.76	444	−114.24	773	−11.66	752	−111.18	735	−115.18	1262	−120.7	1200	−127.8	1118	−112.32
550	618	−121	572	−129.12	449	−118.46	794	−113	766	−120	752	−114.8	1267	−121.5	1211	−121.48	1126	−111.52
600	631	−131	586	−130.66	457	−116.86	842	−123	820	−128.52	809	−124.28	1280	−135.6	1217	−128.78	1145	−117.82

**Table 3:** Network Delay of 0, 1, and 2-m height with (16, 32, 64) bytes Payload Size.

Size	16 Bytes						32 Bytes						64 Bytes					
Height	0 M		1 M		2 M		0 M		1 M		2 M		0 M		1 M		2 M	
Distance	N-D	RSSI	N-D	RSSI	N-D	RSSI	N-D	RSSI	N-D	RSSI	N-D	RSSI	N-D	RSSI	N-D	RSSI	N-D	RSSI
0	120	−22	16	−41.3	8	−48.2	233	−27.4	220	−24.9	208	−42	760	−35.2	749	−56.6	560	−55
50	125	−80.98	84	−99.06	8	−85.86	252	−92.82	240	−78.82	222	−84	775	−99.8	760	−76.86	576	−77.1
100	133	−92.3	91	−108	9	−86.6	260	−99.24	245	−89.9	226	−96.5	779	−105	764	−91	593	−86.7
150	141	−95.6	101	−110	10	−89.8	265	−94.8	255	−86.3	231	−92.8	788	−92.5	768	−93.52	595	−90.7
200	146	−107	111	−110	21	−103	272	−100	267	−92.5	240	−92.4	798	−102	774	−86.44	604	−95
250	157	−103	118	−113	29	−105	279	−100	270	−97.5	281	−107	806	−106.34	776	−104.32	618	−106
300	167	−109	126	−117	33	−113	287	−112	280	−101	287	−111	812	−122	785	−107.9	646	−107
350	176	−115	137	−124	50	−114	293	−112	286	−108	290	−116	820	−120	789	−115.72	655	−115
400	179	−118	142	−127	56	−117	306	−114	298	−107	295	−113	830	−127	794	−118.14	667	−121
450	199	−116	153	−124	60	−118	361	−116	349	−112	324	−118	848	−131	805	−118.8	695	−111.4
500	213	−123	164	−129	64	−114	373	−112	352	−111	335	−115	862	−121	800	−127.8	718	−112.3
550	218	−121	172	−129	69	−118	394	−113	366	−120	352	−115	867	−122	811	−121.84	726	−112
600	230	−131	186	−131	77	−117	442	−123	420	−128.5	409	−124	880	−136	817	128.78	745	−118

**Table 4:** Response Time of 1, 2, and 3 floors with 16, 32, 64 bytes Payload Size.

Size	16 Bytes						32 Bytes						64 Bytes					
Height	1 Floor		2 Floor		3 Floor		1 Floor		2 Floor		3 Floor		1 Floor		2 Floor		3 Floor	
Distance	R-T	RSSI	R-T	RSSI	R-T	RSSI	R-T	RSSI	R-T	RSSI	R-T	RSSI	R-T	RSSI	R-T	RSSI	R-T	RSSI
0	520	−22	40	−41.3	380	−48.16	633	−27.42	620	−24.88	608	−42	1160	−35.13	1149	−56.6	960	−55
50	525	−80.98	484	−99.06	381	−85.86	652	−92.82	640	−78.82	622	−84	1175	−99.8	1160	−76.86	976	−77.1
100	533	−92.3	491	−108	388	−86.58	660	−99.24	645	−89.88	626	−96.48	1179	−104.5	1164	−91	993	−86.72
150	541	−95.6	501	−109.8	390	−89.8	665	−94.84	655	−86.32	631	−92.8	1188	−92.48	1168	−93.52	995	−90.66
200	546	−107	511	−110.4	401	−103	672	−99.98	720	−92.52	640	−92.42	1198	−102	1174	−86.44	1004	−94.96
250	557	−103	518	−112.8	409	−104.5	679	−99.98	675	−97.5	681	−106.7	1206	−106.34	1176	−104.3	1018	−105.7
300	567	−109	526	−117.3	413	−113.4	687	−111.6	680	−100.7	687	−111.2	1212	−121.9	1185	−107.9	1046	−106.5
350	576	−115	537	−124.3	430	−114	693	−111.6	686	−108.3	690	−115.9	1220	−120.2	1189	−115.7	1055	−114.7
400	579	−118	542	−127	436	−117.5	706	−114	698	−106.7	695	−112.9	1230	−127.4	1194	−118.1	1067	−121.2
450	599	−116	553	−124.1	440	−118.2	730	−115.6	749	−111.9	724	−117.7	1248	−131	1205	−118.8	1095	−111.42
500	613	−123	564	−128.8	444	−114.2	773	−111.7	752	−111.2	735	−115.2	1262	−120.7	1200	127.8	1118	−112.32
550	618	−121	572	−129.1	449	−118.5	794	−113	766	−120	752	−114.8	1267	−121.5	1211	121.8	1126	−111.5
600	631	−131	586	−130.7	457	−116.9	842	−123	820	−128.52	809	−124.3	1280	−135.6	1217	−128.8	1145	−117.8

**Table 5:** Network Delay of 1, 2, and 3 Floor with 16, 32, 64 Payload Size.

Size	16 Bytes						32 Bytes						64 Bytes					
Height	1 Floor		2 Floor		3 Floor		1 Floor		2 Floor		3 Floor		1 Floor		2 Floor		3 Floor	
Distance	N-D	RSSI	N-D	RSSI	N-D	RSSI	N-D	RSSI	N-D	RSSI	N-D	RSSI	N-D	RSSI	N-D	RSSI	N-D	RSSI
0	26	−72.3	24	−53.78	23	−14.6	244	−73.2	236	−70.2	237	−30.5	845	−67.2	771	−29.5	738	−40.7
50	33	−75.35	24	−87.2	25	−79.46	258	−74.66	249	−74.66	241	−81.68	858	−74.88	784	−72.16	776	−85.9
100	49	−89.1	47	−89.7	51	−97.2	291	−85.7	281	−80.7	260	−90.3	870	−83.5	788	−80.8	786	−90.4
150	54	−104	49	−115	62	−91.7	321	−93.3	299	−85.3	376	−105	898	−65.6	795	−92.2	790	−97.1
200	59	−109	57	−119	60	−97	347	−101	303	−101	288	−101	908	−99.6	798	−94.7	789	−94.9
250	66	−119	59	−119	59	−91	387	−113	379	−113	362	−99.5	938	−103	805	−102	795	−104
300	70	−116.8	70	−121	65	−107	398	−117	393	−115	365	−105	958	−105	808	−104	805	−102
350	77	−124	73	118	68	−102	425	−113	491	−113	371	−111	973	−113	809	−107	806	−110
400	80	−118	79	−124	72	−112	442	−104	417	−116	408	−107	980	−112	823	−103	817	−118
450	94	−124	85	−128	73	−119	451	−108	422	−108	409	−117	990	−110	825	−121	826	−119
500	104	−124	104	−123	94	−119	516	−121	436	−121	409	−119	1024	−119	831	−113	828	−112
550	152	−131	108	−124	97	−125	519	−126	447	−126	417	−125	1059	−117	840	−118	829	−127
600	198	131.1	124	−128	109	−128	570	−115	470	−115	419	−125	1102	−125	845	−113	830	−121

## Data Availability

No data were used to support this study. We have conducted simulations to evaluate the performance of the proposed protocol. However, any query about the research conducted in this paper is highly appreciated and can be asked from the corresponding authors upon request.
